# 3,3’-Diindolylmethane Enhances Paclitaxel Sensitivity by Suppressing DNMT1-Mediated KLF4 Methylation in Breast Cancer

**DOI:** 10.3389/fonc.2021.627856

**Published:** 2021-06-03

**Authors:** Fenfen Xiang, Zhaowei Zhu, Mengzhe Zhang, Jie Wang, Zixi Chen, Xiaoxiao Li, Tao Zhang, Qing Gu, Rong Wu, Xiangdong Kang

**Affiliations:** ^1^ Laboratory Medicine, Putuo Hospital, Shanghai University of Traditional Chinese Medicine, Shanghai, China; ^2^ General Surgery, Putuo Hospital, Shanghai University of Traditional Chinese Medicine, Shanghai, China

**Keywords:** breast cancer, paclitaxel, sensitivity, KLF4, DNMT1, DIM

## Abstract

Paclitaxel (PTX) is a first-line chemotherapeutic drug for the treatment of breast cancer, but drug resistance seriously limits its clinical use. The aim of the present work was to explore the effect of 3,3’-diindolylmethane (DIM) on PTX sensitivity and its possible mechanism in breast cancer. The expression of Krüppel-like factor 4 (KLF4) and DNA-methyltransferase 1 (DNMT1) in breast cancer tissues were assessed by immunohistochemistry and Western blotting. The methylation of *KLF4* was evaluated by the MassARRAY platform. The lentivirus carrying *KLF4* and *DNMT1* gene or shRNA targeting DNMT1 were used to overexpress KLF4 or knockdown DNMT1 in MCF-7 and T47D breast cancer cells and the role of KLF4 and DNMT1 in regulation of PTX sensitivity was investigated. The effect of PTX on inhibiting the proliferation of MCF-7 and T47D cells was measured by CCK-8 assay. Flow cytometry was used to examine cell apoptosis. The expression of mRNA and protein was evaluated by qRT-PCR and Western blotting analysis, respectively. Our data showed that the expression of DNMT1 was increased, and the methylation level of CpG sites (−148 bp) in the *KLF4* promoter was increased while the KLF4 expression was significantly decreased in breast cancer tissues. Overexpression of KLF4 increased the sensitivity of MCF-7 and T47D cells to PTX. DNMT1 increased the methylation of the KLF4 promoter and decrease the expression of KLF4. Knockdown of DNMT1 increased the sensitivity of MCF-7 and T47D cells to PTX. DIM enhanced the PTX sensitivity of MCF-7 and T47D cells, decreased the expression of DNMT1 and the methylation level of KLF4 promoter, thus increasing the level of KLF4. Furthermore, overexpression of DNMT1 attenuated the effect of DIM on the regulation of PTX sensitivity. Collectively, our data indicated that DNMT1-mediated hypermethylation of *KLF4* promoter leads to downregulation of KLF4 in breast cancer. The level of KLF4 is correlated with the sensitivity of MCF-7 and T47D cells to PTX. DIM could enhance the antitumor efficacy of PTX on MCF-7 and T47D cells by regulating DNMT1 and KLF4.

## Introduction

Breast cancer is the leading cause of cancer-related death among women (1), which remains a challenging task for oncologists. One major obstacle to the successful treatment of patients who receive chemotherapy is drug resistant which resulted in treatment failure. As a well tolerate microtubule stabilizer, PTX is one of the most frequently used anticancer drugs, especially in the treatment of breast cancer; however, its clinical efficacy is limited by drug resistance (2). Therefore, to better understand the drug resistant mechanism of PTX and to enhance its sensitivity are critical for improving the treatment and prognosis of patients.

Krüppel-like factor 4 (KLF4) is a zinc-finger transcriptional factor which is mainly expressed in terminal differentiated epithelial cells and plays regulatory roles in cell proliferation, differentiation and pluripotency (3, 4). Clinically, loss of KLF4 function has been found in various types of cancers and is related to chemotherapeutic resistance (5–7). The low expression of KLF4 in tumor cells was shown to be caused by genetic and epigenetic alterations (8–10). Our preliminary work demonstrated that the expression of KLF4 is related to PTX sensitivity of breast cancer MCF-7 cells. However, the association of KLF4 expression, its functional alteration and PTX sensitivity in breast cancer are not well understood.

Abnormal methylation of genes is one of the epigenetic modifications involved in the development of tumors (11). DNA-methyltransferase (DNMT) is the principle enzyme responsible for maintaining CpG methylation (12), which mediates the transfer of the methyl group from S-adenosylmethionine to 5-methylcytosine base. Methylation of tumor suppressor genes plays critical roles in the pathogenesis of various cancers. It is generally considered that methylation of tumor suppressor gene is equivalent to gene loss or functional mutation (13–15). Therefore, reversing methylation of tumor suppressor genes may be a promising strategy for cancer prevention and treatment (16, 17). It was reported that inhibition of DNMT could enhance chemotherapeutic sensitivity of tumor cells (18, 19). Therefore, it is of great clinical significance to search for specific and low toxic anticancer agents that can reactivate silent cancer suppressor genes by targeting aberrant DNA methylation.

It was known that the bioactive substances in some foods can regulate epigenomes and possess anti-tumor activity. DIM is abundant in cruciferous vegetables, and was showed to have anticancer activities in some tumors, such as breast, cervical and prostate cancers (20, 21). Some clinical trials have been approved for using DIM in patients with premalignant or malignant lesions (22). However, whether DIM could increase the sensitivity of breast cancer cells to PTX and the underlying molecular mechanism remains poorly understood.

In this study, we showed that the KLF4 expression was downregulated in human breast cancer tissues, which was associated with hypermethylation of its promoter region. DIM could enhance PTX sensitivity in MCF-7 and T47D breast cancer cells by reducing the expression of DNMT1 and subsequently reducing the methylation level of *KLF4* and promoting the expression of KLF4. This work provides a new strategy for the improving chemotherapy and sheds a light for further clinical research in breast cancer treatment.

## Materials and Methods

### Tissue Samples

Human breast cancer samples and the adjacent non-malignant breast samples were collected from the Putuo Hospital, Shanghai, China. Before sample collection, all patients signed the informed consent according to the institutional guidelines. This research was granted by the Ethical Review Committee of the Putuo Hospital, Shanghai University of Traditional Chinese Medicine. The procedures of the study were implemented according to the approved guidelines.

### Immunohistochemistry

Immunohistochemistry staining was performed according to the instructions. Briefly, the paraffin-embedded breast tumor tissues were continuously sectioned into sections of 5μm in thickness. The sections were dewaxed and rehydrated. The heat-mediated antigen retrieval was performed by boiling sections in Tris/EDTA at pH 9.0. Primary antibody against KLF4(1:300) or DNMT1(1:200) was used to incubate with tissue sections overnight at 4°C, and then horseradish peroxidase-labeled antibody was used as a secondary antibody for 30 min. The color reaction was carried out with diaminobenzidine as a substrate.

### Cell Cultures

Human breast cancer cell lines (MDA-MB-231, MDA-MB-468, MCF-7 and T47D) were cultured in DMEM (Gibco Industries, Inc.) containing with 10% FBS and maintained at 37°C in a humidified atmosphere with 5% CO_2_.

### Cell Proliferation Assays

MCF-7 and T47D Cells (8000 per well) were seeded onto 96-well plates and incubated overnight. Then, the cells were incubated with different concentrations of PTX (MedCham Express, dissolved in DMSO) alone or in combination with DIM (MedCham Express, dissolved in DMSO). The cells of the control group were treated with DMSO. After 48h of incubation, cell proliferation was measured using the Cell Counting Kit-8 (CCK-8, Dojindo, Japan). The light absorbance was measured at 450 nm.

### Quantitative Real-Time PCR

Total mRNA was isolated from MCF-7 and T47D cells by using TRIzol reagents (Takara Bio, Inc.), and the complementary DNA was synthesized by the PrimeScript RT reagent kit (Takara Bio, Inc.). Reactions were performed at 95°C, 2 min; 95°C, 15 sec and 60°C, 30 sec for 40 cycles in the real time PCR system (StepOne Plus; Applied Biosystems, USA). The relative expression levels of DNMT1 and KLF4 were normalized with GAPDH and calculated by 2^-ΔΔct^ method. The primers used in the research are shown in **Table 1**.

**Table 1:** Nucleotide sequences of primers used for qRT-PCR reactions.

Gene	Forward	Reverse
GAPDH	5’-ATGCTGCCCTTACCCCGG-3’	5’-TTACTCCTTGGAGGCCATGTAGG-3’
DNMT1	5’-CGGCAGACCATCAGGCATTCTAC-3’	5’-CACACCTCACAGACGCCACATC -3’
KLF4	5’-CCTTCAACCTGGCGGACATCAAC -3’	5’-GCTGCTGCGGCGGAATGTAC-3’
Methylation (KLF4)	5’-aggaagagagGTTGTATAGTGTTGGGTATT GTTTT -3’	5’-cagtaatacgactcactatagggagaaggctTTACTATAACAAC TAAATCAACAAACTC-3’

### Western Blotting

Tissues or cells were lysed by the RIPA (Beyotime Institute of Biotechnology, Beijing, China) containing with protease inhibitors. The amounts of protein of each sample were quantified. Protein (50μg) was separated by polyvinylidene gel electrophoresis and transferred onto a polyvinylidene fluoride (PVDF) membrane (Merck Millipore). The membranes were blocked with 5% non-fat milk for 2 h and then hybridized with the KLF4 (CST) or DNMT1 (CST) antibody separately at 4°C overnight and with HRP-conjugated secondary antibodies for 1 h at room temperature. Finally, the immunoreactive bands were determined by enhanced chemiluminescence (Pierce Biotechnology).

### Analysis of Cell Apoptosis

Apoptosis of MCF-7 and T47D breast cancer cells were analyzed using the Annexin V-Phycoerythrin kit (BD Biosciences). After treatment with PTX alone or in combination with DIM for 48 h, MCF-7 and T47D cells were collected and stained with Annexin V-APC (BD Biosciences) in the dark for 20 min. Then, the stained cells were measured by flow cytometry using the Canto (BD Biosciences). The FlowJo software was used to analyze the data.

### Construction of KLF4 Lentiviral Vectors and DNMT1 Vectors

Human KLF4 cDNA was constructed by GenePharma (Shanghai, China), the overexpression of KLF4 and the negative control were named as KLF4-OE and KLF4-NC, respectively. Human DNMT1 cDNA synthesized by Genechem (Shanghai, China) was constructed with CMV-MCS-3FLAG-IRES-EGFP-SV40- neomycin plasmid, the overexpression of DNMT1 was named as DNMT1-OE and the control was named as DNMT1-NC. The shRNA sequence cgACTACATCAAAGGCAGCAA targeted DNMT1 were subcloned with the hU6-MCS-CMV-GFP-SV40-Neomycin plasmid vector, the knockdown of DNMT1was named as DNMT1-KD and the control was named as DNMT1-NC.

### Mass Array Methylation Detection

Genomic DNA was obtained from the breast cancer tissues and cells using a QIAamp DNA kit and the bisulfite conversion reaction was implemented using the EpiTect Bisulfite Kit (Qiagen, Hilden, Germany). PCR primers (**Table 1**) were designed using EpiDesigner. Small RNA fragments with CpG sites were obtained from the PCR products through RNaseA digestion. DNA methylation of CpG was measured using the MassARRAY platform, and the methylation rate was calculated by Epityper software Version 1.0 (Agena, San Diego, CA, USA). The detection and analysis were conducted by the OE Biotech Co., Ltd.

### Statistical Analysis

The analyses of data were used by GraphPad Software, all data were expressed as the mean ± standard deviation from at least three independent experiments. The significance was calculated by Student’s t-test, and p<0.05 was considered as statistically significant difference.

## Results

### The Expression of KLF4 Decreased and Its Promoter Hypermethylated in Breast Cancer

**Figure 1:**
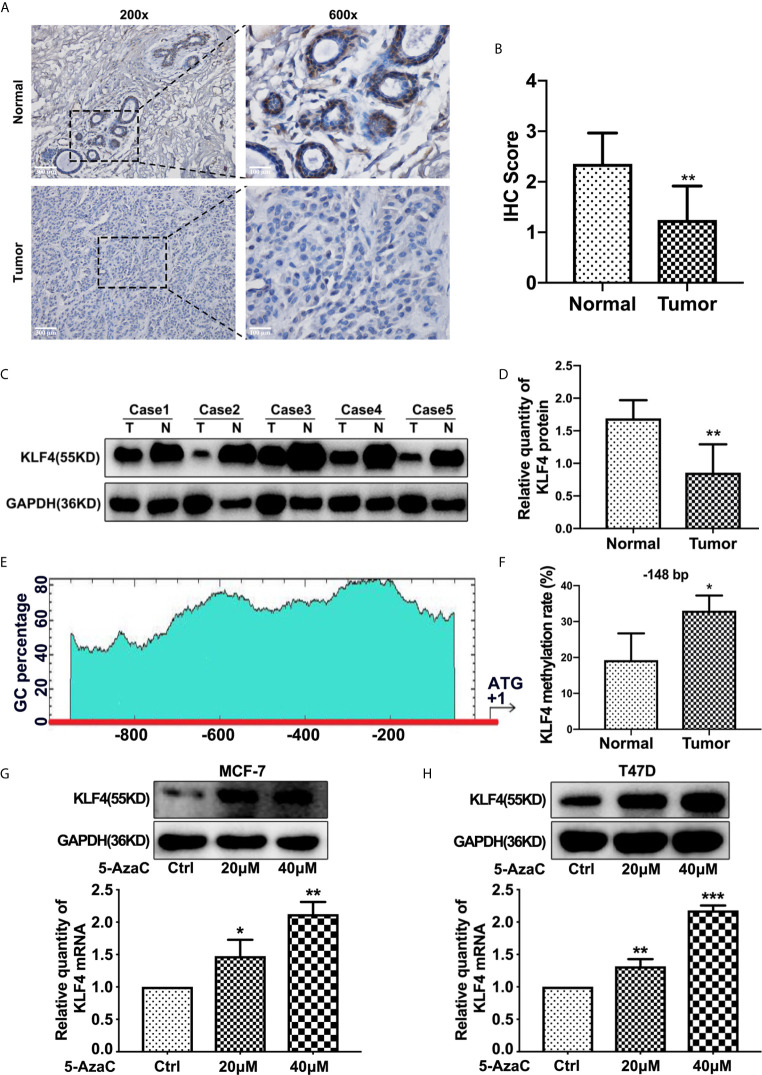
The expression of KLF4 decreased and its promoter hypermethylated in breast cancer **(A)** Immunohistochemical assessment of KLF4 expression in breast cancer tissues. **(B)** Average score of KLF4 immunostaining in breast cancer and adjacent tissues. **(C)** Western blotting analyses of KLF4 protein level in breast cancer and adjacent tissues. **(D)** Relative quantity of KLF4 protein in breast cancer and adjacent tissues. **(E)** The position of CpG sites in the promoter region of *KLF4*. **(F)** Methylation level of the *KLF4* promoter in breast cancer and adjacent tissues. **(G)** Real-time PCR and Western blotting analyses of KLF4 expression levels in MCF-7 cells treated with or without 5-AzaC (20 μmol/L) and 5-AzaC (40 μmol/L) for 48 h. **(H)** Real-time PCR and Western blot analyses of KLF4 expression in T47D cells treated with or without 5-AzaC (20 μmol/L) and 5-AzaC (40 μmol/L) for 48 h. *P<0.05; **P<0.01; ***P<0.001 versus control.

Some previous studies showed that the expression of KLF4 was reduced (23–25), while others reported that KLF4 expression was increased in breast cancer tissues (26, 27). To determine the level of KLF4 in breast cancer tissues, we determined the KLF4 expression in 50 breast cancer and adjacent normal tissues by immunohistochemical (IHC) staining. The results showed that the expression of KLF4 was decreased in breast cancer tissues (**Figures 1A, B**). Also, the Western blot analysis showed that the KLF4 level was significantly lower in breast tumor tissues compared with normal tissues (**Figures 1C, D**).

The expression of KLF4 is regulated by the DNA methylation status of its promoter. Therefore, we explored whether the decreased expression of KLF4 was associated with the DNA methylation level of its promoter. The methylation level of *KLF4* promoter was measured in breast carcinoma tissues (n = 5) and adjacent normal tissues (n = 5) by the MassArray methylation assay. The selected regions for the bisulfifite sequencing and the CpG island in *KLF4* promoter region are shown in **Figure 1E**. The results showed that the methylation level of the CpG sites (−148 bp) in the *KLF4* promoter was significantly higher in breast tumor tissues than in adjacent normal tissues (**Figure 1F**).

Additionally, to investigate whether the downregulation of KLF4 was caused by the hypermethylation of its promoter, MCF-7 and T47D cells were treated with 5-Aza-2′-Deoxycytidine (5-AzaC), an inhibitor of DNA methyltransferase. The results showed that 5-AzaC significantly increase the expression level of KLF4 in MCF-7 and T47D cells (**Figures 1G, H**). This result confirmed that downregulation of KLF4 was caused by the hypermethylation of its promoter.

### The Expression of KLF4 Was Associated With PTX Sensitivity in Breast Cancer Cells

**Figure 2:**
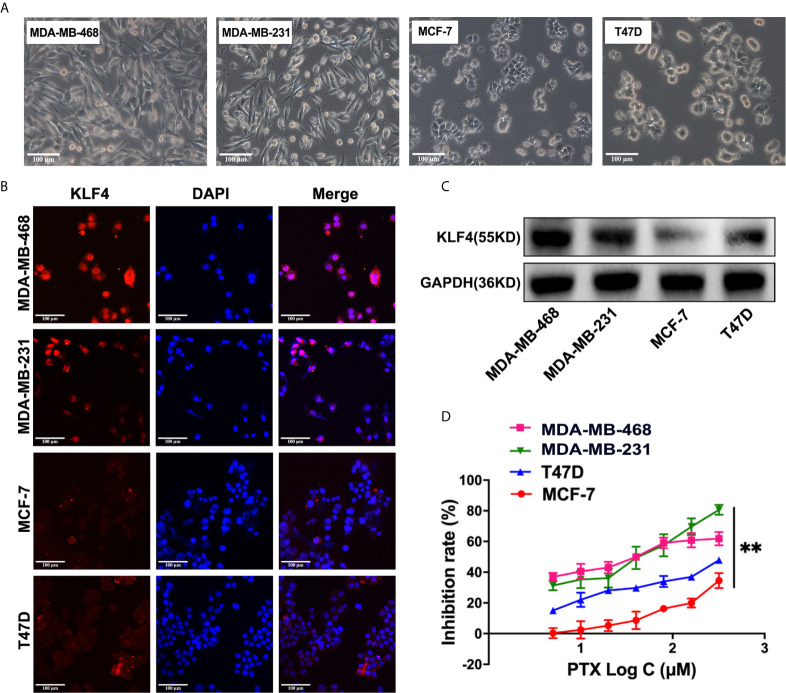
The expression of KLF4 was associated with PTX sensitivity in breast cancer. **(A)** Morphological images of cell lines. **(B)** Immunofluorescence analyses of KLF4 protein expression in MBA-MD-468, MBA-MD-231, MCF-7 and T47D cells. **(C)** Western blotting analyses of KLF4 protein expression in MBA-MD-468, MBA-MD-231, MCF-7 and T47D cells. **(D)** The inhibition of MBA-MD-468, MBA-MD-231, T47D and MCF-7 cell growth by PTX treatment. **P<0.01 versus control.

The KLF4 expression was evaluated in different breast cancer cells, including the MDA-MB-468, MDA-MB-231, MCF-7 and T47D breast cancer cells (**Figure 2A**) by immunofluorescent staining and Western blotting. It was found that the expression of KLF4 was different in the different breast cancer cells (**Figures 2B, C**). To investigate the correlation between the KLF4 level and cell sensitivity to PTX, we evaluated the cell growth inhibition after treatment with PTX in these four cell lines. The results showed that the MDA-MB-468 and MDA-MB-231 cells had higher KLF4 level and are more sensitive to PTX than those of MCF-7 and T47D cells, that had lower KLF4 level and are less sensitive to PTX (**Figure 2D**). The results suggested that the level of KLF4 was related to the PTX sensitivity in breast cancer cells.

### Overexpression of KLF4 Increased the Sensitivity of Breast Cancer Cells to PTX

**Figure 3:**
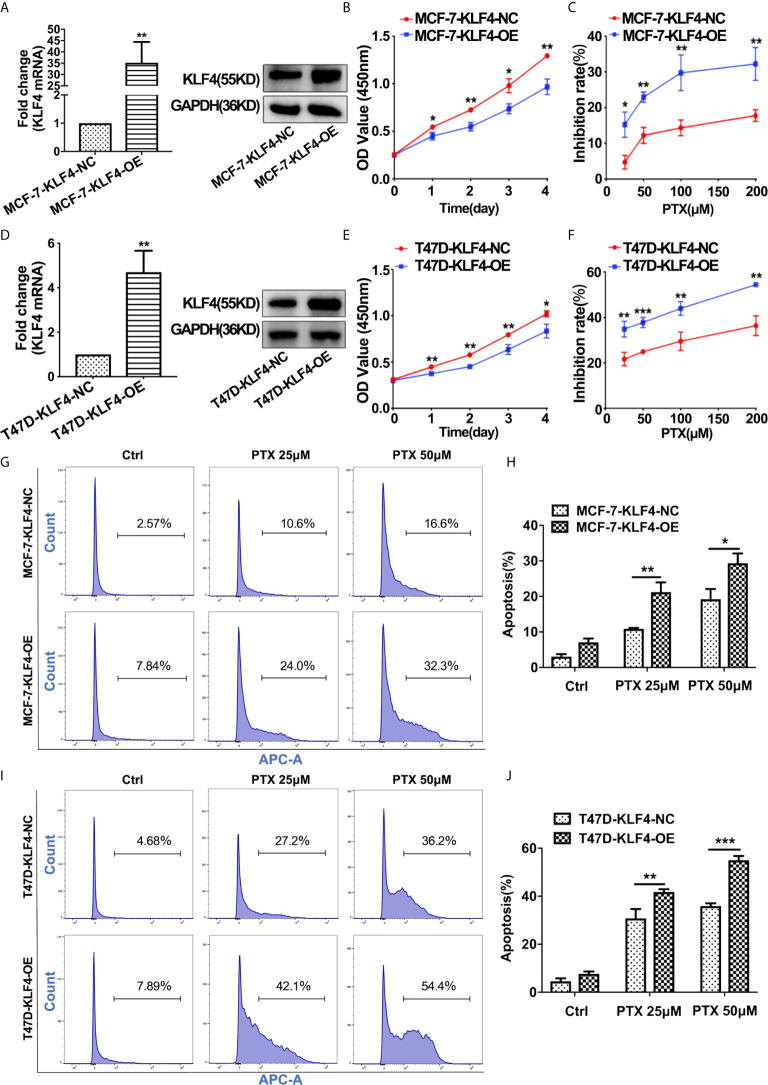
Overexpression of KLF4 increased the sensitivity of breast cancer cells to PTX. **(A)** Real-time PCR and Western blot analyses of KLF4 expression after transfection with the KLF4 overexpressing lentivirus in MCF-7 cells. **(B)** Growth curve of MCF-7 cells after overexpression of KLF4. **(C)** The inhibition of MCF-7 cell proliferation by PTX after KLF4 overexpression. **(D)** Real-time PCR and Western blot analyses on the expression of KLF4 after transfection with the KLF4 overexpressing lentivirus in T47D cells. **(E)** Growth curve of T47D cells after overexpression of KLF4. **(F)** The inhibition of T47D cell proliferation by PTX after KLF4 overexpression. **(G)** Apoptosis in MCF-7 cells treated with PTX after KLF4 overexpression. **(H)** Apoptotic cell percentage of MCF-7 cells treated with PTX. **(I)** Apoptosis of T47D cells treated with PTX after KLF4 overexpression. **(J)** Apoptotic cell percentage of T47D cells treated with PTX. *P<0.05; **P<0.01; ***P< 0.001 versus control.

In order to determine whether KLF4 mediates PTX sensitivity in breast cancer cells, the lentiviral vector containing *KLF4* were used to overexpress KLF4 in MCF-7 and T47D breast cancer cells. The qRT-PCR and Western blot analysis showed that the KLF4 level was significantly higher in MCF-7 and T47D cells transfected with the *KLF4* lentiviral vector compared with the control cells (**Figures 3A, D**). The proliferation was decreased in of MCF-7 and T47D cells after overexpressing KLF4 (**Figures 3B, E**). Further, the proliferation was significantly inhibited by PTX in MCF-7 and T47D cells after overexpressing KLF4 compared with the control cells (**Figures 3C, F**). These results suggested that overexpression of KLF4 could increase the sensitivity of MCF-7 and T47D cells to PTX.

In order to investigate whether KLF4 could mediate apoptosis of MCF-7 and T47D cells after the PTX treatment, we tested apoptotic changes in MCF-7 and T47D cells using Annexin V-7AAD after PTX treatment for 48h. The results showed that the percentage of apoptotic cells was significantly increased in the cells overexpressed KLF4 (**Figures 3G–J**). This result suggested that the KLF4 overexpression was related to PTX-induced apoptosis in MCF-7 and T47D cells.

### DNMT1 Expression Was Negatively Correlated With KLF4 Expression and PTX Sensitivity in Breast Cancers

**Figure 4:**
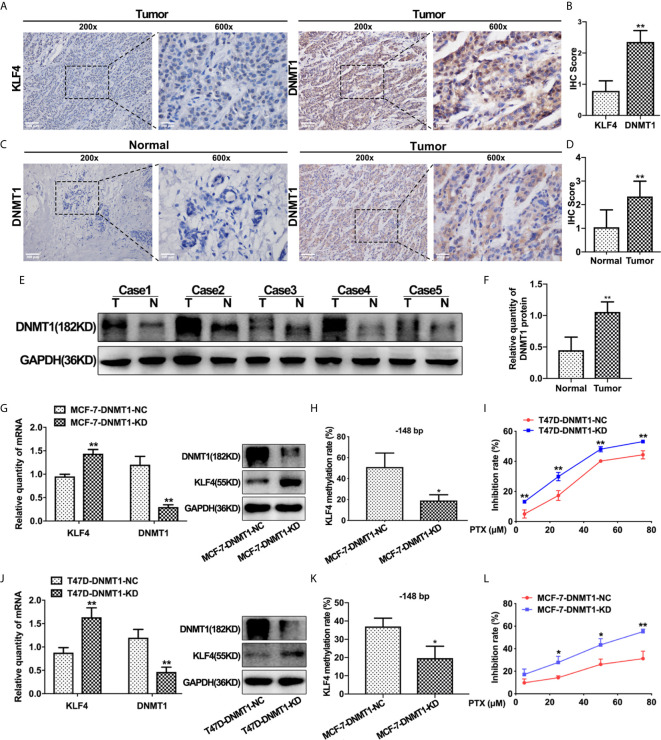
DNMT1 expression was negatively correlated with KLF4 expression and PTX sensitivity in breast cancers. **(A)** Immunohistochemical assessment of KLF4 and DNMT1 levels in breast cancer tissues. **(B)** Average score of KLF4 and DNMT1 immunostaining in breast cancer tissues. **(C)** Immunohistochemical assessment of DNMT1 expression in breast cancer and adjacent tissues. **(D)** Average score of DNMT1 immunostaining in breast cancer and adjacent tissues. **(E)** Western blotting analyses of DNMT1 protein expression in breast cancer and adjacent tissues. **(F)** Relative quantity of DNMT1 protein expression in breast cancer and adjacent tissues. **(G)** Real-time PCR and Western blot analysis of DNMT1 expression level in MCF-7 cells after transfection with the DNMT1 interference plasmid. **(H)** Methylation level of the KLF4 promoter in MCF-7 cells after transfection with the DNMT1 interference plasmid. **(I)** The viability of MCF-7 cells treated with PTX (5μM, 25μM, 50μM and 75μM) after transfection with the DNMT1 interference plasmid. **(J)** Real-time PCR and Western blot analysis of DNMT1 expression in T47D cells after transfection with the DNMT1 interference plasmid. **(K)** Methylation level of the KLF4 promoter in T47D cells after transfection with the DNMT1 interference plasmid. **(L)** The viability of T47D cells treated with PTX (5μM, 25μM, 50μM and 75μM) after transfection with the DNMT1 interference plasmid. *P<0.05; **P<0.01 versus control.

In order to find out the possible reason underlying hypermethylation of KLF4 promoter in breast cancer, we detected the expression of DNMT1 in these 50 breast carcinoma and adjacent normal tissues by IHC analysis. The results showed that the expression of DNMT1 was lower in breast normal tissues with relatively high KLF4 expression, while DNMT1 was significantly increased in breast cancer tissues with relatively low KLF4 expression. These results indicated that DNMT1 expression was negatively correlated with KLF4 level in breast cancer (**Figures 4A, B**). Additionally, DNMT1 expression levels were significantly higher in carcinoma tissues than in adjacent tissues (**Figures 4C, D**). Similarly, the Western blot test showed that DNMT1 protein was significantly higher in carcinoma tissues (n = 5) than in adjacent normal tissues (n = 5) (**Figures 4E, F**).

To further confirm that the expression of KLF4 is regulated by DNA methyltransferase, DNMT1 shRNA was used to knockdown DNMT1 in MCF-7 and T47D cells. It was found that KLF4 mRNA and protein expression were significantly increased (**Figures 4G, J**), and the methylation of the CpG sites (−148 bp) in the *KLF4* promoter was significantly decreased (**Figures 4H, K**) upon decreasing DNMT1 expression. Additionally, the proliferation of MCF-7 and T47D cells was significantly inhibited by PTX after down-regulation of DNMT1 compared with that of the control cells (**Figures 4I, L**), suggesting that knockdown of DNMT1 could enhance the sensitivity of MCF-7 and T47D cells to PTX. These results indicated that DNMT1 and KLF4 play a critical effect in regulating the sensitivity of PTX in breast cancer cells.

### DIM Enhanced PTX Sensitivity in Breast Cancer

**Figure 5:**
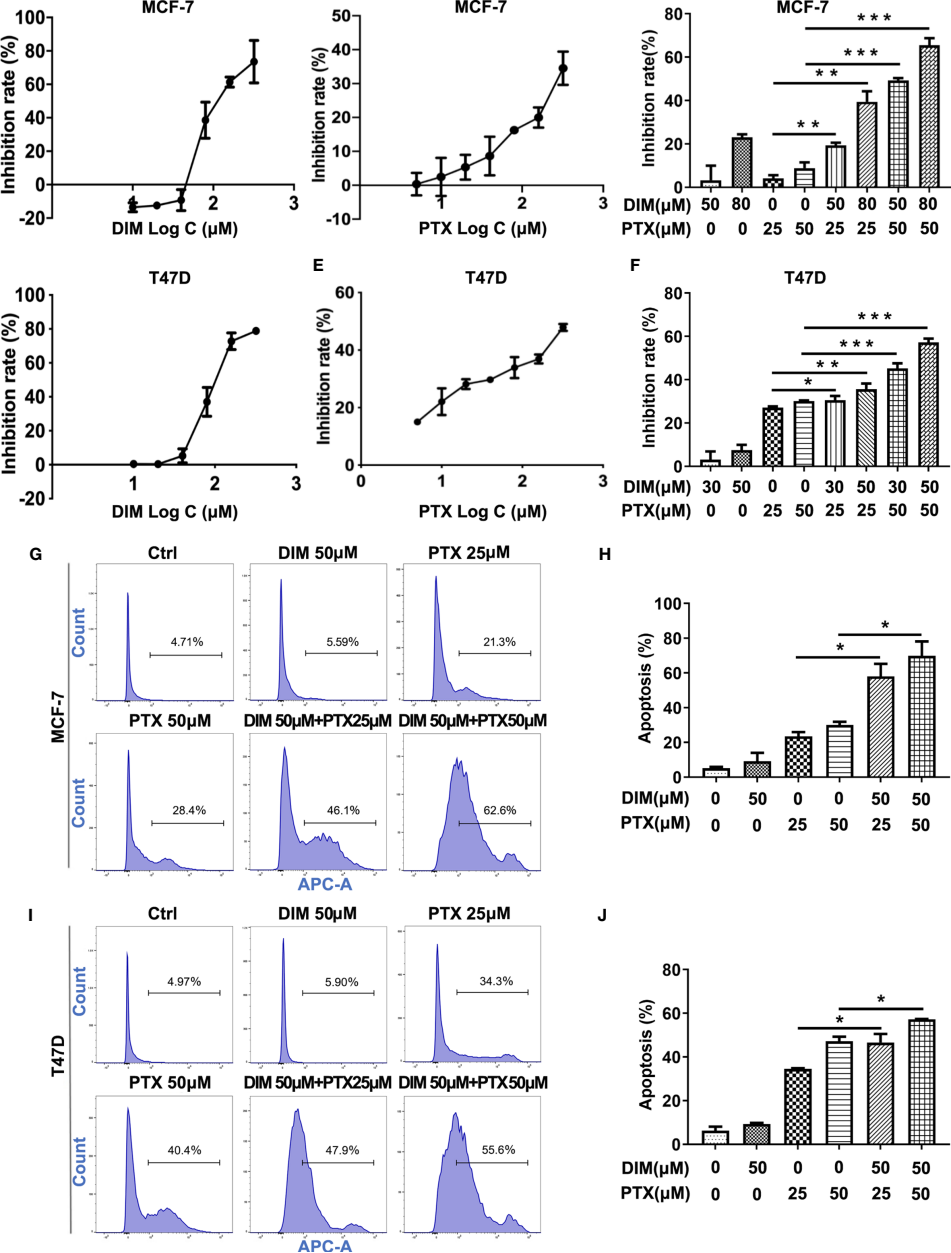
DIM enhanced PTX sensitivity in breast cancer cells. **(A)** The viability of MCF-7 cells after DIM (10μM, 20μM, 40μM, 80μM, 160μM and 320μM) treatment. **(B)** The viability of MCF-7 cell after PTX (5μM,10μM, 20μM, 40μM, 80μM, 160μM and 320μM) treatment. **(C)** The viability of MCF-7 cells treated with PTX after DIM pretreatment. **(D)** The viability of T47D cells after DIM (10μM, 20μM, 40μM, 80μM, 160μM and 320μM) treatment. **(E)** The viability of T47D cells after PTX (5μM,10μM, 20μM, 40μM, 80μM, 160μM and 320μM) treatment. **(F)** The viability of T47D cells treated with PTX after DIM pretreatment. **(G)** Apoptosis in MCF-7 cells treated with PTX after DIM pretreatment. **(H)** Percentage of apoptosis of MCF-7 cells treated with PTX after DIM pretreatment. **(I)** Apoptosis in T47D cells treated with PTX after DIM pretreatment. **(J)** Percentage of apoptosis of MCF-7 cells treated with PTX after DIM pretreatment. *P<0.05; **P<0.01; ***P< 0.001 versus control.

Furthermore, we explored whether DIM could enhance the effect of PTX on MCF-7 and T47D breast cancer cells. The concentrations of DIM used were chosen to be 50 μM and 80 μM for the MCF-7 cells, and 30 μM and 50 μM for T47D cells, and the CCK-8 results showed that DIM at a low toxic concentration significantly enhanced the efficacy of PTX in inhibition of cell growth in MCF-7 (**Figures 5A–C**) and T47D cells (**Figures 5D–F**). To confirm whether DIM can change the apoptotic rate of MCF-7 and T47D cells after PTX treatment, we tested the apoptotic changes of MCF-7 and T47D cells by Annexin V. It was found that the rate of apoptotic cells was increased significantly in cells treated with DIM in combination with PTX (**Figures 5G–J**). These results suggested that DIM can enhance PTX sensitivity in breast cancer cells.

### DIM Enhanced PTX Sensitivity by Inhibiting DNMT1 and Downregulating KLF4 Methylation

**Figure 6:**
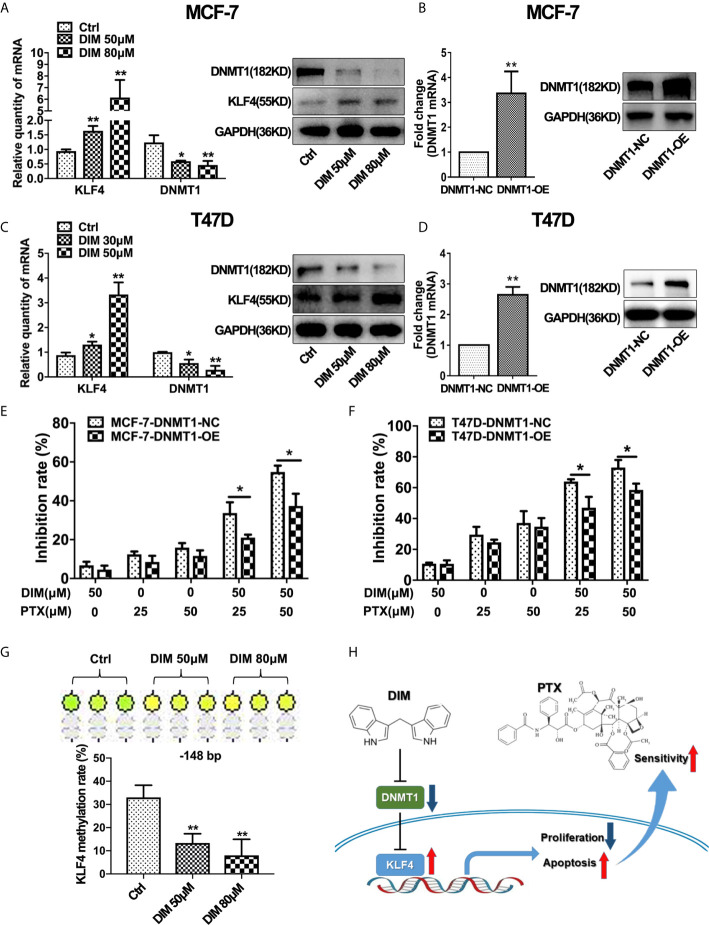
DIM enhanced PTX sensitivity by inhibiting DNMT1 and downregulating KLF4 methylation. **(A)** Real-time PCR and Western blotting analyses of KLF4 and DNMT1 expression in MCF-7 cells treated with or without DIM (50 μM or 80 μM) for 48 h. **(B)** Real-time PCR and Western blot analyses of DNMT1 expression after transfection with the DNMT1 overexpressing plasmid in MCF-7 cells. **(C)** Real-time PCR and Western blotting analyses of KLF4 and DNMT1 expression in T47D cells treated with or without DIM (30 μM or 50 μM) for 48 h. **(D)** Real-time PCR and Western blotting analyses of DNMT1 expression in T47D cells transfected with the DNMT1 overexpressing plasmid. **(E)** The viability of MCF-7 cells treated with PTX and DIM after overexpressing DNMT1. **(F)** The viability of T47D cells treated with PTX and DIM after overexpressing DNMT1. **(G)** Methylation levels of the *KLF4* gene in MCF-7 cells treated with or without DIM (50 μM or 80 μM) for 48 h. **(H)** Schematic model of DIM in enhancing PTX sensitivity by regulating the expression of KLF4 and DNMT1. *P<0.05; **P<0.01 versus control.

Studies have suggested that DIM plays an important role in regulating the expression of DNMT1 (28). Therefore, we evaluated whether DIM affects DNMT1 and KLF4 expression. Real-time PCR and Western blotting analysis revealed that DIM could significantly decrease the DNMT1 expression and increase the KLF4 expression in MCF-7 and T47D breast cancer cells (**Figures 6A, C**). To demonstrate the effect of DIM on enhancing PTX sensitivity through targeting DNMT1, we used a virus vector carrying DNMT1 to increase the expression of DNMT1 in MCF-7 and T47D cells. qRT-PCR and Western blot analysis showed that the DNMT1 levels were significantly higher in MCF-7 and T47D cells transfected with the DNMT1 vector compared with the control cells (**Figures 6B, D**). It was found that overexpression of DNMT1 reduced the inhibitory effect of proliferating MCF-7 and T47D cells after DIM and PTX combined treatment (**Figures 6E, F**). This result suggested that DIM was unable to enhance the sensitivity of MCF-7 and T47D cells to PTX when the expression of DNMT1 was increased. We further investigated whether DIM regulates the methylation level of *KLF4* promoter in breast cancer cells, and the results showed that compared with normal cells, the methylation level of CpG sites (−148 bp) in the *KLF4* promoter was decreased in breast cancer MCF-7 cells after treatment with DIM (**Figure 6G**). These results showed that DIM enhanced PTX sensitivity by inhibiting DNMT1 expression and subsequently upregulating the KLF4 level in the breast cancer cells (**Figure 6H**).

## Discussion

PTX is commonly used in the treatment of breast cancer, but its clinical effect is limited due to the development of drug resistance. Therefore, there is an urgent need to find agents that can be used for effectively reversing PTX resistance in breast cancer. Here, we demonstrated that DNMT1 promoted the methylation of *KLF4* promoter and thus decreased KLF4 expression, which was correlated with the PTX sensitivity in breast cancer. In addition, DIM could effectively increase the sensitivity of MCF-7 and T47D breast cancer cells to PTX by downregulating KLF4 methylation through inhibiting DNMT1.

The role and expression of KLF4 in tumors are controversial. Some studies reported that KLF4 is upregulated in breast cancer tissues (26, 27), while others found that KLF4 is downregulated (23–25). In our study, we found that the expression of KLF4 was significantly decreased in breast cancer tissues. Studies have demonstrated that the expression of KLF4 was regulated by the DNA methylation status of its promoter (28). To determine whether the decrease in KLF4 expression is due to the increase in methylation level, we investigated the methylation status of the *KLF4* promoter in breast cancer tissues, and found that the methylation level of specific CpG site (−148 bp) in the *KLF4* promoter played a critical role in the regulation of KLF4 expression.

KLF4 plays a key role in chemotherapeutic treatments by regulating gene expression that required for signaling pathways (29). Furthermore, studies have shown that KLF4 affected chemoresistance of some chemotherapeutic drugs (30). However, the effect of KLF4 in PTX sensitivity of breast cancer cells has not been reported. Here, we demonstrated that the KLF4 expression was correlated with PTX sensitivity and that breast cancer cells with a lower KLF4 level were less sensitive to PTX. Moreover, we demonstrated that overexpression of KLF4 can increase PTX-induced inhibition of cell growth and promoted apoptosis of MCF-7 and T47D cells, which indicates that restoring KLF4 expression of breast cancer cells could be an effective way to overcome PTX resistance.

DNMT1 is required in maintaining CpG methylation and silencing of various tumor suppressor genes. Increased DNMT1 level often leads to the increased tumorigenesis and elevated DNMT1 expression was associated with PTX resistance (31, 32). Studies have shown that DNMT1 reduced the expression of KLF4 by promoting methylation of its promoter (33, 34). In our present study, we found that DNMT1 expression increased in breast cancer tissues. Furthermore, after knockdown DNMT1 expression in MCF-7 and T47D breast cancer cells, the expression of KLF4 increased, and the sensitivity of breast cancer cells to PTX significantly increased. Our results demonstrated that DNMT1 plays a critical role in breast cancer development and PTX sensitivity.

It has been proved that DNA demethylation drug is effective in adjuvant therapy of some solid cancers and hematological malignancies (35). KLF4 is a major target of demethylation drugs in enhancing the PTX sensitivity of breast cancer. Therefore, a drug that can inhibit DNMT1 expression and promote the expression of KLF4 would considerably improve the status of PTX resistance in the clinical treatment of breast cancer. Dietary DIM is a natural compound and has been proved to be a low toxic substance that can potentially inhibit the expression of DNMT1 (28), and can be used in the prevention and treatment of clinical cancer. Our study demonstrated that dietary DIM can significantly increase the sensitivity of MCF-7 and T47D cells to PTX. To further investigate the mechanism of action of DIM in enhancing PTX sensitivity, we determined the expression levels of DNMT1 and KLF4 in breast cancer MCF-7 and T47D cells after treatment with DIM. We found that DIM can increase the KLF4 expression and decrease the DNMT1 expression level in MCF-7 and T47D cells.

In summary, our findings demonstrate that the expression of DNMT1 and the methylation level of *KLF4* promoter increased, while the expression of KLF4 decreased in breast cancer tissues. Knockdown of DNMT1 can enhance PTX sensitivity by increasing KLF4 expression. DIM can effectively decrease the expression of DNMT1 and the methylation level of KLF4 in breast cancer cells, and then promote the expression of KLF4. Our study suggests that DIM can increase the sensitivity of PTX and therefore, combination of DIM and PTX could be an effective strategy to overcome drug resistance in breast cancer cells.

## Data Availability Statement

The original contributions presented in the study are included in the article. Further inquiries can be directed to the corresponding authors.

## Ethics Statement

The studies involving human participants were reviewed and approved by Ethical Review Committee of the Putuo Hospital, Shanghai University of Traditional Chinese Medicine. The patients/participants provided their written informed consent to participate in this study.

## Author contributions 

KX, WR and XF conceived and designed the project. XF, ZZ and ZM performed the experiments and acquired the data. WJ and ZZ collected tissue specimens. CZ and GQ analyzed the data. XF and ZM wrote the article. LX and ZT participated in writing the article. WR and KX commented and edited the article. All authors contributed to the article and approved the submitted version.

## Funding

This work was supported by the National Natural Science Foundation of China (No. 81703878), the Shanghai Rising Stars of Medical Talent Youth Development Program and the Putuo Hospital, Shanghai University of Traditional Chinese Medicine (No. 2020373B).

## Conflict of Interest

The authors declare that the research was conducted in the absence of any commercial or financial relationships that could be construed as a potential conflict of interest.
